# Genetic Modifiers of Oral Nicotine Consumption in *Chrna5* Null Mutant Mice

**DOI:** 10.3389/fpsyt.2021.773400

**Published:** 2021-11-04

**Authors:** Erin Meyers, Zachary Werner, David Wichman, Hunter L. Mathews, Richard A. Radcliffe, Joseph H. Nadeau, Jerry A. Stitzel

**Affiliations:** ^1^Institute for Behavioral Genetics, University of Colorado Boulder, Boulder, CO, United States; ^2^Skaggs School of Pharmacy and Pharmaceutical Sciences, University of Colorado Anschutz Medical Campus, Aurora, CO, United States; ^3^Maine Medical Center Research Institute, Scarborough, ME, United States; ^4^Department of Integrative Physiology, University of Colorado Boulder, Boulder, CO, United States

**Keywords:** chromosome substitution strains, two-bottle choice, nicotinic acetylcholine receptor, mapping, knockout

## Abstract

The gene *CHRNA5* is strongly associated with the level of nicotine consumption in humans and manipulation of the expression or function of *Chrna5* similarly alters nicotine consumption in rodents. In both humans and rodents, reduced or complete loss of function of *Chrna5* leads to increased nicotine consumption. However, the mechanism through which decreased function of *Chrna5* increases nicotine intake is not well-understood. Toward a better understanding of how loss of function of *Chrna5* increases nicotine consumption, we have initiated efforts to identify genetic modifiers of *Chrna5* deletion-dependent oral nicotine consumption in mice. For this, we introgressed the *Chrna5* knockout (KO) mutation onto a panel of C57BL/6J-Chr#^A/J^/NAJ chromosome substitution strains (CSS) and measured oral nicotine consumption in 18 CSS and C57BL/6 (B6) mice homozygous for the *Chrna5* KO allele as well as their *Chrna5* wild type littermates. As expected, nicotine consumption was significantly increased in *Chrna5* KO mice relative to *Chrna5* wildtype mice on a B6 background. Among the CSS homozygous for the *Chrna5* KO allele, several exhibited altered nicotine consumption relative to B6 *Chrna5* KO mice. Sex-independent modifiers were detected in CSS possessing A/J chromosomes 5 and 11 and a male-specific modifier was found on chromosome 15. In all cases nicotine consumption was reduced in the CSS *Chrna5* KO mice relative to B6 *Chrna5* KO mice and consumption in the CSS KO mice was indistinguishable from their wild type littermates. Nicotine consumption was also reduced in both *Chrna5* KO and wildtype CSS mice possessing A/J chromosome 1 and increased in both KO and wild type chromosome 17 CSS relative to KO and wild type B6 mice. These results demonstrate the presence of several genetic modifiers of nicotine consumption in *Chrna5* KO mice as well as identify loci that may affect nicotine consumption independent of *Chrna5* genotype. Identification of the genes that underlie the altered nicotine consumption may provide novel insight into the mechanism through which *Chrna5* deletion increases nicotine consumption and, more generally, a better appreciation of the neurobiology of nicotine intake.

## Introduction

It has become evident that nicotinic acetylcholine receptors (nAChRs) that contain the α5 subunit play a critical role in the risk for nicotine dependence. Human studies repeatedly have found an association between genetic variants in *CHRNA5*, the gene that codes for the nAChR α5 subunit, nicotine dependence and other nicotine dependence relevant phenotypes (1–7). In fact, a recent genome-wide meta-analysis reported that a missense (amino acid changing) SNP in *CHRNA5* (rs16969968) exhibited the strongest association with cigarettes per day of all tested SNPs (*P* = 1.2 × 10^−278^) (7).

Studies in rodents further support the role of *Chrna5* in nicotine-related behaviors. For example, a few early studies demonstrated that *Chrna5* played a role in sensitivity to the acute effects on nicotine in mice (8, 9). More recent studies using rodent models have provided further insight into the role of *Chrna5* in nicotine dependence. For example, Fowler et al. (10) found that *Chrna5* knockout (KO) mice, unlike wildtype controls, did not reduce their responding for i.v. nicotine self-administration as the unit dose increased. At the highest nicotine dose tested, *Chrna5* KO mice self-administered five times more nicotine than did their wildtype controls. Similarly, Jackson et al. (11) reported that *Chrna5* KO mice exhibited conditioned place preference at higher doses of nicotine than did wildtype controls and Wilking and Stitzel (12) as well as Bagdas et al. (13) showed that *Chrna5* KO mice consume more nicotine via oral administration, especially at higher nicotine concentrations, relative to wildtype littermates.

It is important to note that the non-synonymous mutation in *CHRNA5* that is highly associated with increased risk for nicotine use, including nicotine consumption, in humans leads to a reduction in function of α5-containing nAChRs (3, 14–17). In other words, reduced function of α5-containing nAChRs is associated with various measures of nicotine use in humans, including increased nicotine consumption, and increased nicotine self-administration and reward in rodents. The sum of these studies suggests that understanding the molecular mechanism through which reduced/loss of function of *Chrna5* leads to increased nicotine consumption may lead to a better understanding of the neurobiology of nicotine use.

One approach that can be used to gain insight into the mechanism through which loss of function of *Chrna5* increases nicotine consumption is to identify genetic modifiers of the increased nicotine consumption caused by *Chrna5* deletion in mice. In essence, a modifier gene is a gene that has one or more alleles that suppresses, enhances or in some other way alters the outcome of an allele or alleles of another gene known to have a phenotypic effect (in this case *Chrna5* deletion increasing nicotine consumption) often without having a measurable effect on the phenotype itself (18, 19). Presumably, modifier genes work by altering a molecular process that is essential for producing the phenotype caused by the allele of the target gene. As a result, the identification of modifier genes can provide novel insight into the underlying molecular processes important for producing the target gene-dependent phenotype. Although there are many approaches to identify genetic modifiers, we utilized a panel of chromosome substitution strains (CSS) to provide evidence for the existence of modifiers of *Chrna5* KO-dependent nicotine consumption. Chromosome substitution strains (CSS) are a panel of strains that share a common genetic background but differ from one another for a single whole chromosome that comes from a donor strain. A full panel of mouse CSS consists of twenty-two strains, one strain for each of the 19 donor strain autosomes, one each for the donor strain sex chromosomes and one strain harboring the mitochondrial genome from the donor strain [for a review of CSS see (20, 21)]. By parsing the donor strain genome into single chromosomes on the host genome background, CSS have proven to be a very powerful tool for identifying and mapping quantitative trait loci (QTL) (22).

To utilize the CSS to identify chromosomes that harbor modifiers that alter the effect of *Chrna5* deletion on nicotine consumption, we bred the *Chrna5* KO allele into the panel of C57BL/6J-Chr#^A/J^/NaJ CSS (23). For this CSS panel, the host background strain is C57BL/6J (B6), an inbred strain that consumes the most nicotine relative to other tested strains, and the donor strain is A/J, a strain that is amongst the lowest nicotine consuming strains (24, 25). Following introgression of the *Chrna5* KO allele onto the panel of C57BL/6J-Chr#^A/J^/NaJ CSS, oral nicotine consumption was measured in each CSS and the B6 reference strain. Both wildtype and *Chrna5* KO mice from each CSS and B6 were tested in order to identify A/J chromosomes possessing modifiers of *Chrna5* KO-dependent an independent nicotine consumption.

## Materials and Methods

### Animals

#### Housing Conditions

All housing and experimental conditions for the mice utilized in this study were approved by the Institutional Animal Care and Utilization Committee (IACUC) at the University of Colorado Boulder and were compliant with the guidelines for animal care and use mandated by the NIH and the Guide for the Care and Use of Laboratory Animals (8th Ed.). Mice were maintained on a standard 12 h light/dark cycle (lights on at 07:00), and food (Envigo Teklad 2914 irradiated rodent diet, Harlan, Madison, WI) and water were available *ad libitum*.

#### Generation of CSS^*Chrna*5*KO*^ Strains

**Figure 1:**
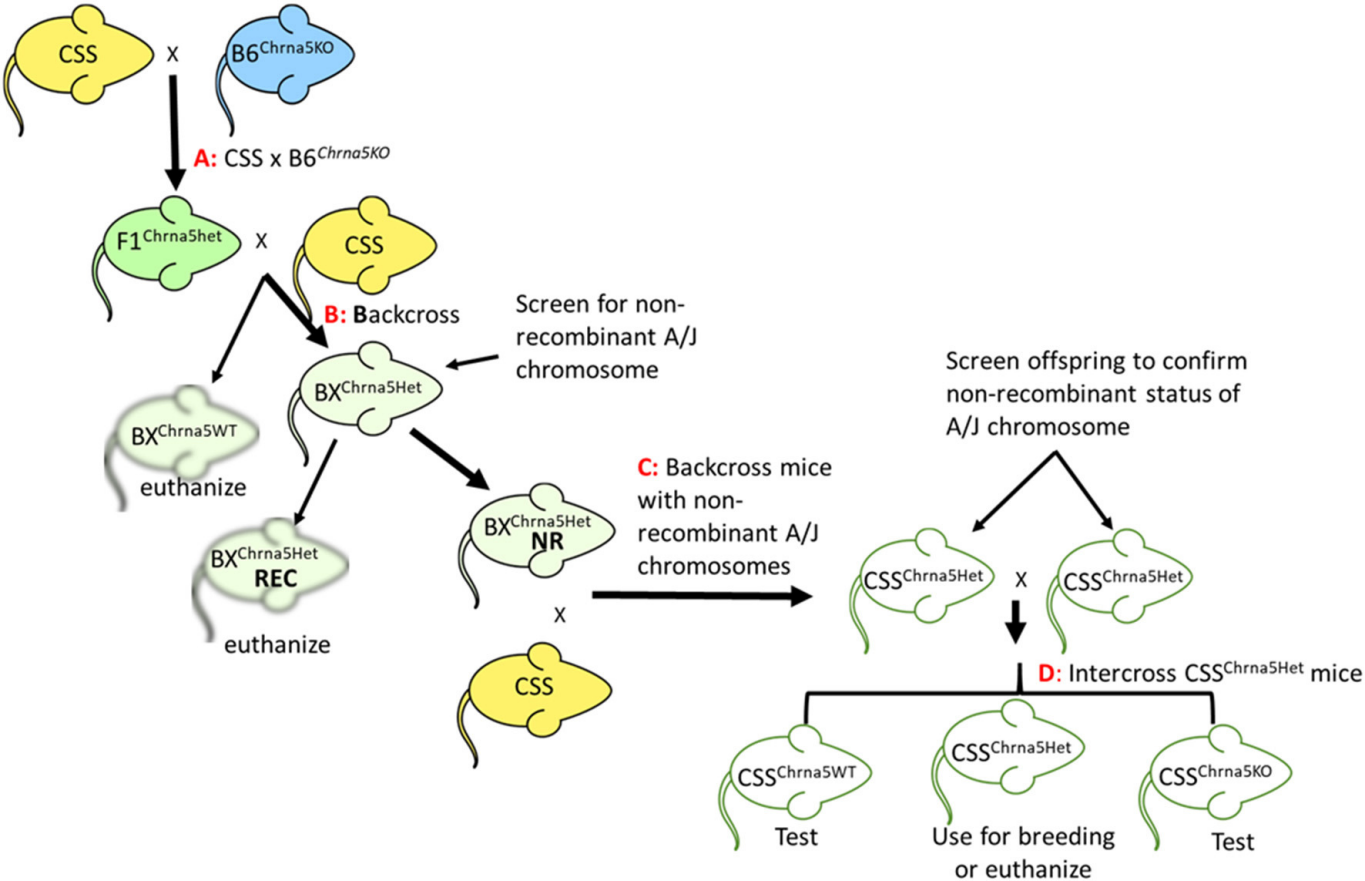
Schematic of the breeding strategy used to generate CSS possessing the *Chrna5* null mutation. **(A)** Cross between a parental CSS and *Chrna5*KO mice on a B6 genetic background (B6^*Chrna5*KO^). **(B)** Backcross of F1 mice heterozygous for the *Chrna5* KO allele (F1^Chrna5*het*^) generated by cross **(A)** to CSS parental strain. **(C)** Backcross offspring from backcross **(B)** to parental CSS. Only backcross offspring that were determined to be non-recombinant for the appropriate A/J chromosome (BX^Chrna5*het*^ NR) were used. Backcross animals that were recombinant for the appropriate A/J chromosome (BX^Chrna5*het*^ REC) were euthanized. **(D)** Intercross between littermates that possessed a non-recombinant A/J chromosome and were heterozygous for the *Chrna5* KO allele (CSS^Chrna5*het*^). CSS mice WT for *Chrna5* (CSS^Chrna5*WT*^) or homozygous for the *Chrna5* KO allele (CSS^*Chrna5*KO^) resulting from intercross **(D)** were used for the two-bottle choice test.

Chromosome substitution strains C57BL/6J-Chr#^A^/J/NaJ (20, 23), hereafter referred to as CSS, for all chromosomes except Y and the mitochondrial genome, were purchased from the Jackson Laboratory (JAX, Bar Harbor, Maine) and imported to the University of Colorado Boulder. The JAX strain IDs for the CSS are continuous from #004379 to #004398. *Chrna5* null mutant mice were originally obtained from Dr. Mariella de Biasi (9) (MGI:3040917) and have been maintained on a C57BL/6J (B6) background at the University of Colorado Boulder for over 20 years. To minimize genetic drift for mice in our colony that are maintained on a B6 background, new JAX C57BL/6J mice (strain #000664) have been introduced into the University of Colorado Boulder vivarium every 2–3 years. The *Chrna5* null mutation was introgressed into each of the CSS strains using a multi-step process (Figure 1). Initially, each CSS was crossed to B6^*Chrna5*KO^ mice. These mice were then backcrossed to the appropriate parental CSS strain. Offspring of this backcross that were heterozygous for the *Chrna5* null mutation were then screened for the strain-specific non-recombinant A/J chromosome using a series of SNP alleles (see Supplementary Table 1 for all SNPs used for screening). SNP genotyping was performed by CD Genomics (Shirley NY). Of note, screening the CSS parental strains (prior to any backcrossing) identified 10 SNPs that were homozygous for a B6 allele suggesting incomplete introgression of the A/J chromosome. Six of these SNPs were located near the centromere or telomere of the introgressed chromosome indicating some residual B6 chromosome appears to be present on a few A/J chromosomes (1, 11, 15, 16, 17, and X). Four of these centromeric/telomeric SNPs are in regions previously reported to exhibit incomplete introgression of the A/J chromosome (26). Of the four non-centromeric, non-telomeric SNPs, two have conflicting genotype information and the remaining two SNPs have not been evaluated in more than a handful of strains. Therefore, any interpretation from these SNPs should be done with caution.

Following SNP screening, a minimum of five mice per CSS that were verified as possessing a non-recombinant A/J chromosome and heterozygous for the *Chrna5* null mutation were then backcrossed again to the parental CSS strain and offspring were again genotyped to verify the non-recombinant status of the A/J chromosome. Offspring of this cross that were heterozygous for the *Chrna5* null mutation were then intercrossed to produce wildtype (WT), heterozygous and homozygous mutant mice. Initial litters of these mice were also screened to verify non-recombinant status of the appropriate A/J chromosome. Only WT and homozygous mutant mice (CSS#^*Chrna5*KO^) were used for phenotypic assessments. Of the 20 CSS, CSS4, and CSS13 did not breed well so insufficient animals were produced for testing. In addition, *Chrna5* is located on chromosome 9 and we were not able to introgress the region of chromosome 9 centromeric to *Chrna5* into a CSS. Therefore, chromosome 9 from the CSS9^*Chrna5*KO^ line only has been introgressed for the A/J chromosome telomeric to *Chrna5*.

#### Two-Bottle Choice Test (2BCT)

Both female and male WT and null mutant mice were tested. The mice were tested between 3 and 6 months of age. Two weeks prior to starting the 2BCT, mice were moved from ventilated cage racks and housed in standard static cages. This 2-week acclimation period was necessary for the mice to adjust to using water bottles with sipper tubes since their water source in the ventilated cages was a hydropac (Lab Products LLC, Seaford, DE). Preliminary experiments indicated that without this acclimation period to adjust to sipper tubes, 2BCT results were inconsistent. Following the 2-week acclimation period, mice were individually housed and provided 2 glass tubes (PYREX™ 150 mm reusable Borosilicate Glass Tubes, Thermo-Fisher) filled with tap water and fitted with standard ball bearing-free sipper tubes. After 4 days, the tubes were replaced with one tube containing water and the other containing water supplemented with 100 μg/ml nicotine free base (Sigma Aldrich, St. Louis, MO). Four days later, the nicotine concentration was increased to 200 μg/ml and 4 days after that, the nicotine concentration was increased to 300 μg/ml. After an additional 4 days, the experiment was terminated and the animals were euthanized. Throughout the experiment, bottles were rotated daily to minimize any side preference shown by the mice and mice were weighed at the beginning and end of each 4-day period. Fresh tubes and solutions were used for each 4-day testing period. To estimate fluid consumption, tubes were weighed at the beginning and end of each 4-day period or more frequently if the fluid levels needed to be replenished before the end of any given test period. Two cages with tubes but no mice were used to estimate fluid loss due to evaporation, tube rotation and cage handling. The average fluid loss from these “dummy” cages was subtracted from the fluid amount measured from the experimental cages to correct for non-specific fluid loss.

#### Data Analysis

All statistical analyses were performed in SPSS version 27 or Graphpad Prism version 9. Because a mixed model ANOVA analysis detected main effects of both strain and sex on total fluid consumed and average weight across each of the 4-day consumption trials, the measure that was used to assess nicotine consumption was μg of nicotine consumed per ml of total fluid consumed (μg/ml). The μg/ml measure of nicotine consumption eliminates any bias caused by differences in fluid consumption between strain or sex by normalizing nicotine intake to per ml of fluid consumed and also eliminates weight as a factor in the intake calculation. For analyses involving repeated measures, a mixed model ANOVA was utilized that included nicotine concentration as a within subject factor and *Chrna5* genotype, sex and strain as between subject factors. For non-repeated measures, a multi-factorial General Linear Model was used. For both types of analysis, initial analyses for the main effects of sex and strain were performed. Because main effects of sex and strain were observed for all measures, each strain was assessed for sex differences and if sex differences were detected, *Chrna5* genotype was analyzed separately by sex. When no effect of sex was detected for a strain, *Chrna5* genotype analyses were collapsed on sex.

Within-*Chrna5* genotype analyses also were performed separately in *Chrna5* WT and *Chrna5* null mutant mice to assess whether nicotine consumption differed between each CSS strain and the relevant reference B6 strain (B6 WT for CSS WT and B6^*Chrna5*KO^ for CSS^*Chrna5*KO^). Total μg/ml of nicotine consumed was analyzed via a multi-factorial ANOVA of sex and strain. Welch's ANOVA was used for all subsequent one-way analyses. For those strains in which a main effect of sex was observed, data were assessed and reported separately by sex, otherwise analyses were collapsed on sex. A false discovery rate (FDR) (27) of 0.05 was used to identify CSS that significantly differed from the relevant B6 reference strain.

## Results

In order to screen for potential genetic modifiers that alter the effect of *Chrna5* deletion on oral nicotine intake, the *Chrna5* null mutation was introgressed onto a panel of 20 B6 x A/J chromosome substitution strains (CSS) (20, 23) as described in the methods. These *Chrna5* null mutant-harboring strains are designated as CSS^*Chrna*5*KO*^. Due to poor breeding, two of the CSS, CSS4 and CSS13, were excluded from the study.

Because the most common measure used to assess oral nicotine intake is dose and dose is dependent upon the amount of fluid intake as well as the weight of the mice, the CSS panel was assessed for whether there was a main effect of strain on either fluid intake or animal body weight across the study. Results indicated main effects of strain on both fluid consumption [*F*_(18, 919)_ = 24.58, *P* = 2.58 × 10^−66^] and body weight [*F*_(18, 919)_ = 21.75, *P* = 2.59 × 10^−61^], respectively. Therefore, the main measure we used to assess nicotine consumption was μg of nicotine consumed per ml of total fluid drank (μg/ml) which should minimize any confound caused by strain-dependent differences in total fluid intake and weight.

**Figure 2:**
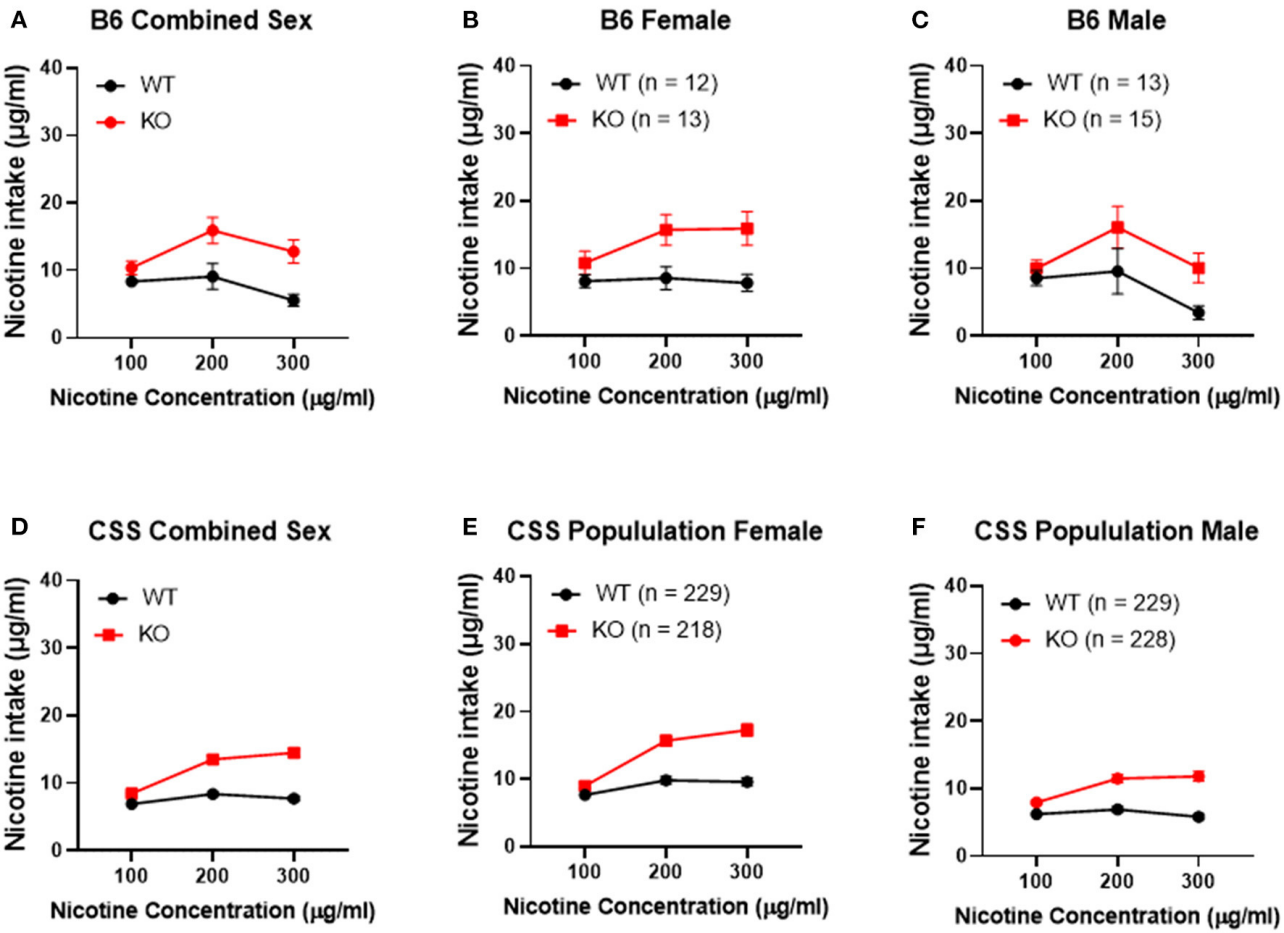
Effect of *Chrna5* genotype on nicotine consumption in B6 and chromosome substitution strain (CSS) mice. Deletion of *Chrna5* leads to increased nicotine consumption in B6 background mice (*P* = 0.003). Although no main effect of sex was observed in B6 mice, combined sex **(A)** as well as data for each sex [female **(B)** and male **(C)**] are shown for comparison. For the entire population of CSS mice, there is a main effect of *Chrna5* genotype as well as a main effect of sex. The impact of *Chrna5* genotype collapsed on sex **(D)** and in female CSS **(E)** (*P* = 1.76 × 10^−10^) and male CSS **(F)** (*P* = 3.43 × 10^−11^) is shown. Data represent mean ± SEM.

To confirm that *Chrna5* genotype impacts nicotine consumption as previously reported (12, 13), female and male B6 background mice that were homozygous for the *Chrna5* null mutation as well as their wildtype (WT) littermates were tested in an ascending two-bottle choice test for oral nicotine consumption at nicotine concentrations 100, 200, and 300 μg/ml. Results (Table 1 and Figures 2A–C) confirmed a main effect of *Chrna5* genotype on nicotine consumption on a B6 background (*P* = 0.003) with *Chrna5* null mutant mice consuming significantly more nicotine than WT mice. There was no main effect of sex and no genotype by sex interaction. Examination of the entire population of tested CSS mice (Table 1 and Figures 2D–F) also indicated a main effect of genotype (*P* = 5.9 × 10^−20^) as well as sex (*P* = 1.6 × 10^−10^) but no genotype x sex interaction. Consistent with B6 background mice, CSS mice harboring the *Chrna5* null mutation, on average, consuming more nicotine than CSS mice that were WT for *Chrna5*.

**Table 1:** Statistics for population comparisons.

**Population tested**	***Chrna5* genotype**	**Sex**	**Genotype x sex interaction**	**Strain**	**Strain x genotype interaction**
B6	*F*_(1, 49)_ = 9.81, *P =* 0.003	*F*_(1, 49)_ = 0.792, *P =* 0.378	*F*_(1, 49)_ = 0.099, *P =* 0.754	NA	NA
CSS collapsed on strain	*F*_(1, 900)_ = 87.72, *P =* 5.9 × 10^−20^	*F*_(1, 900)_ = 41.85, *P =* 1.6 × 10^−10^	*F*_(1, 900)_ = 0.772, *P =* 0.380	NA	NA
CSS by strain	*F*_(1, 832)_ = 116.2, *P =* 1.88 × 10^−25^	*F*_(1, 832)_ = 50.71, *P =* 2.33 × 10^−12^	*F*_(1, 832)_ = 0.949. *P =* 0.330	*F*_(17, 832)_= 13.82, *P =* 5.02 × 10^−35^	*F*_(17, 832)_ = 2.02, *P =* 0.006
B6 vs. CSS *Chrna5* KO genotype	NA	*F*_(1, 18)_ = 24.29, *P =* 1 × 10^−6^	NA	*F*_(1, 18)_ = 8.13, *P =* 1.1 × 10^−18^	NA
B6 vs. CSS *Chrna5* WT genotype	NA	*F*_(1, 18)_ = 29.22, *P =* 1.1 × 10^−7^	NA	*F*_(1, 18)_ = 5.93, *P =* 6.7 × 10^−13^	NA

After confirming that *Chrna5* genotype impacts nicotine intake in the CSS, it was then determined whether individual strain impacted nicotine consumption and/or the effect of *Chrna5* genotype on consumption. In order to simplify the phenotype, total μg/ml of nicotine consumed across the experiment (the sum of nicotine consumed at 100, 200, and 300 μg/ml) was used for the analysis. For total μg/ml of nicotine consumed, main effects of strain (*P* = 5.02 × 10^−35^), genotype (*P* = 1.88 × 10^−25^) and sex (*P* = 2.33 × 10^−12^) as well as a strain x genotype interaction (*P* = 0.006) were observed (Table 1).

**Figure 3:**
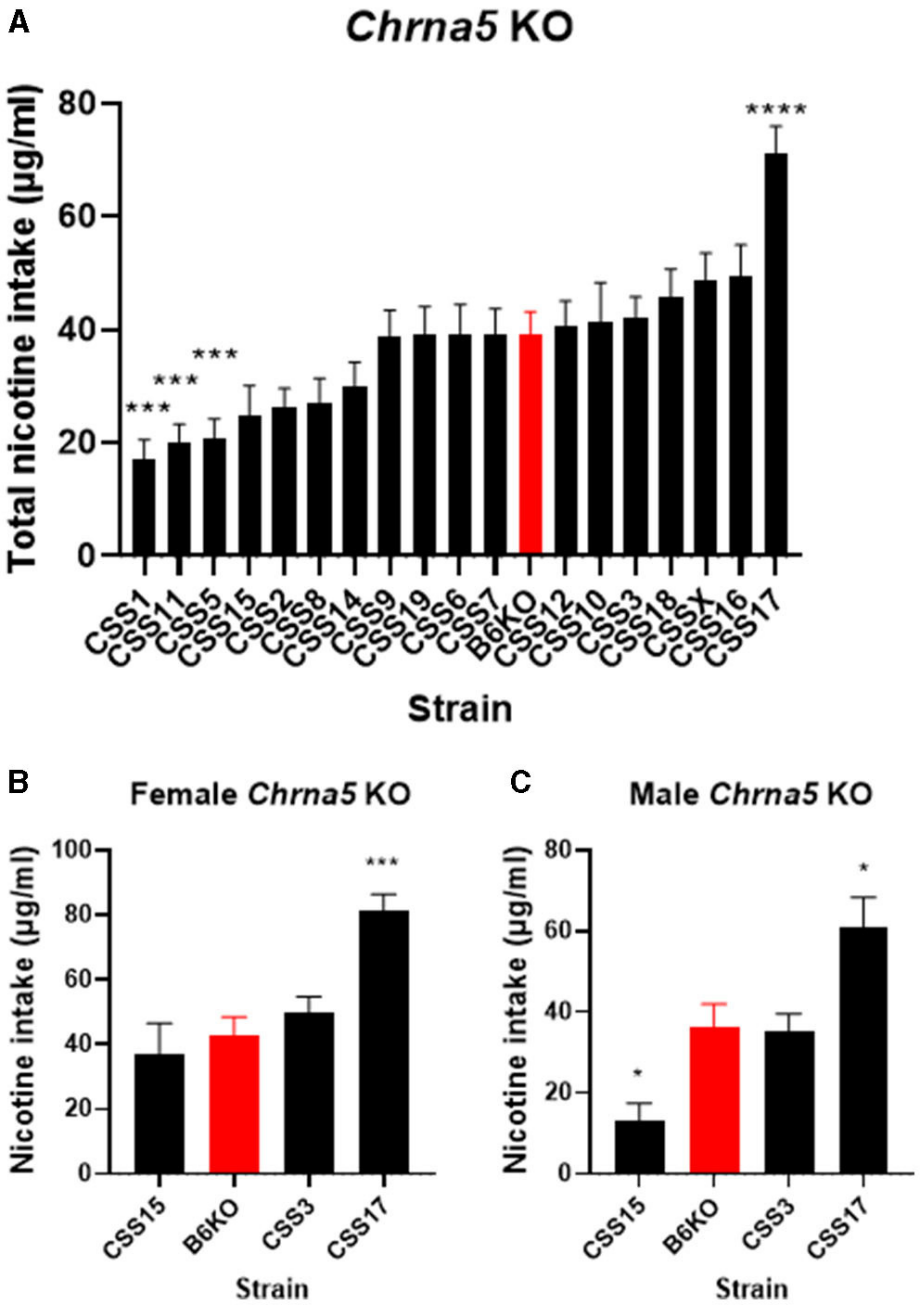
Within *Chrna5* KO genotype comparison of nicotine consumption between individual CSS^*Chrna5*KO^ and B6^*Chrna5*KO^ mice (B6KO). **(A)** Shows total nicotine consumption for all strains collapsed on sex **(A)**. For those strains in which there was a main effect of sex, data are shown separately for female **(B)** and male **(C)** animals. ^*^q < 0.05, ^***^q < 0.005, ^****^q <5 × 10^−5^ for pairwise comparison of nicotine consumption between CSS^*Chrna5KO*^ and B6^*Chrna*5KO^. Data shown represent mean ± SEM. *N* = 9–16 per strain per sex.

The significant strain x genotype interaction on nicotine intake suggests the possibility of strain-specific modifiers that alter the effect of *Chrna5* deletion on nicotine consumption. Because the main goal of this study is to identify CSS that harbor modifiers of the effect of *Chrna5* deletion on nicotine intake, the level of nicotine consumption in each CSS homozygous for the *Chrna5* null mutation (CSS^*Chrna5*KO^) was compared to B6 mice homozygous for the null mutation (B6^*Chrna5*KO^) in a within genotype analysis (Table 1 and Figure 3). Initial analysis indicated main effects of strain (*P* = 1.1 × 10^−18^) and sex (*P* = 1 × 10^−6^). Due to the main effect of sex, the CSS^*Chrna5*KO^ strains were individually assessed for the effect of sex on nicotine intake (Table 2). Of the 18 CSS^*Chrna5*KO^ strains, only three, CSS3^*Chrna5*KO^, CSS15^*Chrna5*KO^ and CSS17^*Chrna5*KO^, exhibited sex-dependent differences in nicotine intake. Therefore, these three strains were assessed separately by sex while the remaining strains were collapsed on sex for analysis. Among the CSS^*Chrna5*KO^ with no main effect of sex, pairwise comparisons between B6*Chrna5*KO mice and each CSS*Chrna5*KO strain using an FDR = 0.05 indicated that CSS1^*Chrna5*KO^ (*q* < 0.005), CSS5^*Chrna5*KO^ (*q* < 0.005), and CSS11^*Chrna5*KO^ (*q* < 0.005) consumed less nicotine then did B6^*Chrna5*KO^ mice (Figure 3A). Due to the discovery nature of this study, the FDR threshold was raised to 0.1 to identify suggestive differences. However, no additional strains were detected with this elevated threshold. For the strains with a main effect of sex, nicotine consumption in CSS3^*Chrna5*KO^ mice did not differ from B6^*Chrna5*KO^ mice for either sex while nicotine consumption in CSS15^*Chrna5*KO^ mice was reduced compared to B6^*Chrna5*KO^ in males but not females (female *q* > 0.05; male *q* < 0.05) and increased relative to B6^*Chrna5*KO^ for both sexes in CSS17^*Chrna5*KO^ mice (female *q* < 0.005; male *q* < 0.05) (Figures 3B,C). Increasing the FDR to 0.1 did not identify any additional strains with suggestive sex-dependent effects.

**Table 2:** Within *Chrna5* genotype analysis of sex effects.

**Strain**	**Main effect of sex in *Chrna5*** **KO mice**	**Main effect of sex in *Chrna5*** **WT mice**
B6KO	*F*_(1, 28)_ = 0.566, *P =* 0.459	*F*_(1, 23)_ = 0.263, *P =* 0.613
CSS1	*F*_(1, 22)_ = 2.37, *P =* 0.138	*F*_(1, 22)_ = 3.85, *P =* 0.062
CSS2	*F*_(1, 26)_ = 0.584, *P =* 0.452	*F*_(1, 22)_ = 0.903, *P =* 0.352
CSS3	*F*_(1, 23)_ = 5.39, *P =* 0.030	*F*_(1, 21)_ = 8.32, *P =* 0.009
CSS5	*F*_(1, 24)_ = 1.96, *P =* 0.175	*F*_(1, 24)_ = 5.96, *P =* 0.023
CSS6	*F*_(1, 22)_ = 2.94, *P =* 0.100	*F*_(1, 24)_ = 0.003, *P =* 0.955
CSS7	*F*_(1, 22)_ = 2.18, *P =* 0.289	*F*_(1, 24)_ = 0.141, *P =* 0711
CSS8	*F*_(1, 23)_ = 1.27, *P =* 0.271	*F*_(1, 23)_ = 0.646, *P =* 0.430
CSS9	*F*_(1, 22)_ = 0.015, *P =* 0.903	*F*_(1, 22)_ = 3.08, *P =* 0.093
CSS10	*F*_(1, 19)_ = 1.54, *P =* 0.230	*F*_(1, 24)_ = 0.477, *P =* 0.496
CSS11	*F*_(1, 22)_ = 0.018, *P =* 0.893	*F*_(1, 22)_ = 2.99, *P =* 0.098
CSS12	*F*_(1, 21)_ = 0.608, *P =* 0.444	*F*_(1, 23)_ = 4.6, *P =* 0.043
CSS14	*F*_(1, 22)_ = 3.36, *P =* 0.08	*F*_(1, 22)_ = 8.42, *P =* 0.008
CSS15	*F*_(1, 29)_ = 5.24, *P =* 0.030	*F*_(1, 29)_ = 7.81, *P =* 0.009
CSS16	*F*_(1, 25)_ = 1.31, *P =* 0.263	*F*_(1, 28)_ = 6.62, *P =* 0.016
CSS17	*F*_(1, 22)_ = 5.40, *P =* 0.030	*F*_(1, 26)_ = 0.666, *P =* 0.442
CSS18	*F*_(1, 22)_ = 0.554 *P =* 0.465	*F*_(1, 22)_ = 0.462 *P =* 0.504
CSS19	*F*_(1, 22)_ = 2.38, *P =* 0.137	*F*_(1, 22)_ = 2.78, *P =* 0.110
CSSX	*F*_(1, 22)_ = 1.39, *P =* 0.252	*F*_(1, 22)_ = 6.15, *P =* 0.021

**Figure 4:**
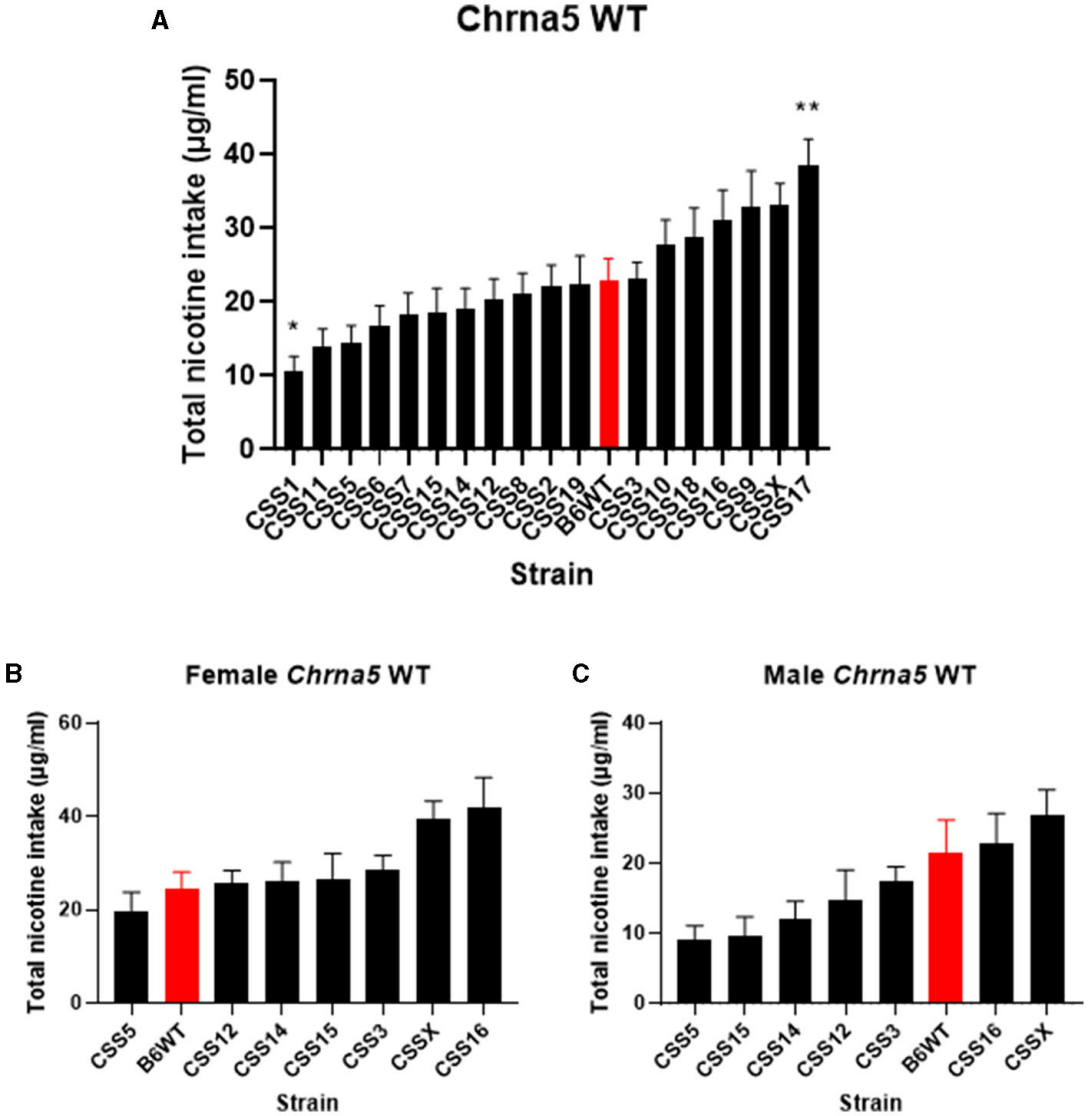
Within *Chrna5* WT genotype comparison of nicotine consumption between individual CSS^*Chrna5*WT^ and B6^*Chrna5*WT^ mice (B6WT). **(A)** Shows total nicotine consumption for all strains collapsed on sex **(A)**. For those strains in which there was a main effect of sex, data are shown separately for female **(B)** and male **(C)** animals. ^*^q < 0.05, ^**^q < 0.01 for pairwise comparison of nicotine consumption between CSS^*Chrna*5WT^ and B6^*Chrna*5WT^. Data shown represent mean ± SEM. *N* = 12–17 per strain per sex.

The effect of the individual CSS on nicotine intake in mice homozygous for the *Chrna5* null mutation could be specific to the *Chrna5* deletion or could be a main effect of the CSS on nicotine consumption (i.e., independent of *Chrna5* genotype). To establish whether the effect of strain on nicotine consumption was specific to mice possessing the *Chrna5* null mutation or a general effect on nicotine intake, nicotine consumption was assessed in all of the CSS strains possessing the WT allele of *Chrna5* and compared to B6 WT mice (Table 1 and Figure 4). This analysis identified main effects of strain (*P* = 6.7 × 10^−13^) and sex (*P* = 1.1 × 10^−7^). Again, due to the main effect of sex, individual CSS strains were evaluated for significant sex effects (Table 2). Results indicated that WT CSS3, CSS5, CSS12, CSS14, CSS15, CSS16, and CSSX exhibited sex-dependent differences in nicotine intake and, therefore, were analyzed by individual sex while the remaining strains were collapsed on sex. For those strains collapsed on sex, CSS1 WT (*q* < 0.05) consumed less nicotine than did B6 WT mice while CSS17 WT (*q* < 0.05) drank more nicotine than did B6 WT mice (Figure 4A). For those CSS in which there was a main effect of sex, none differed from B6 background mice for either sex at either an FDR of 0.05 or 0.1 (Figures 4B,C).

**Figure 5:**
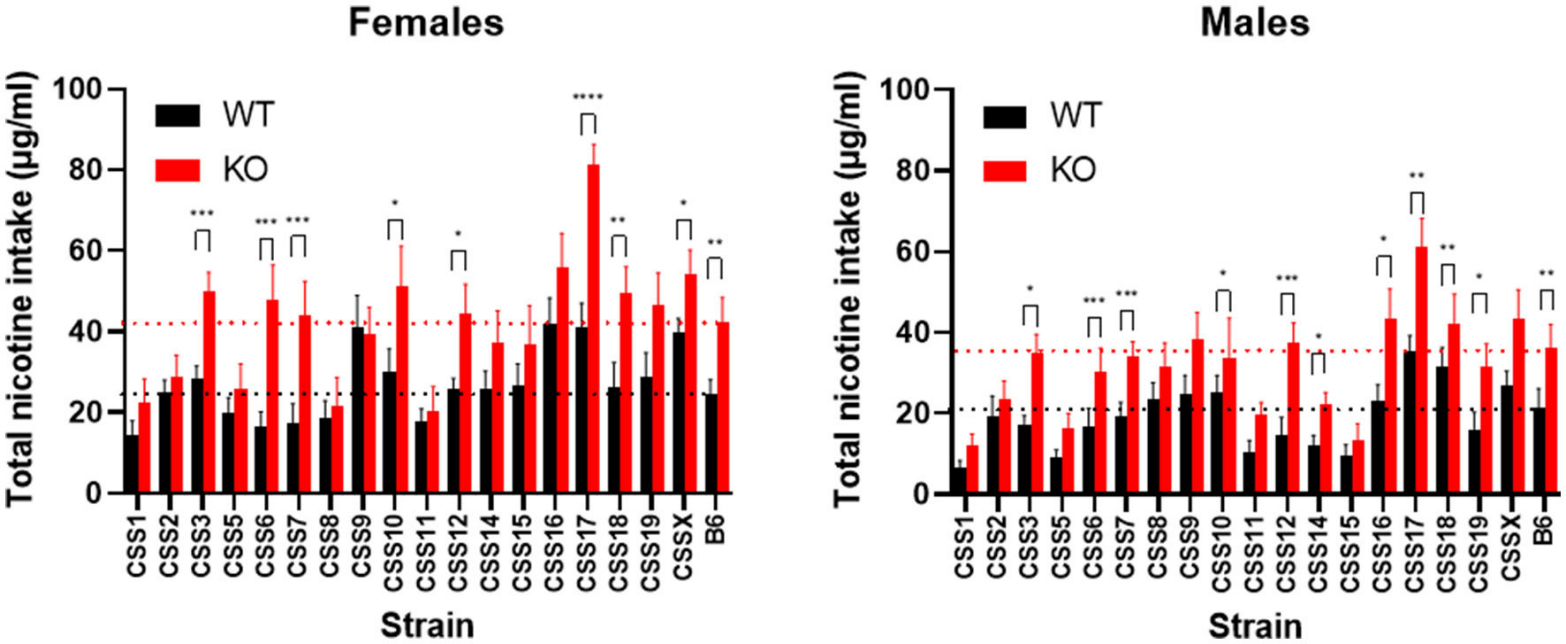
Comparison of nicotine consumption between WT and *Chrna5* KO mice for each CSS. Black dotted line indicates mean intake from the B6 WT strain and the red dotted line represents mean nicotine intake from B6 *Chrna5*KO mice. ^*^p < 0.05, ^**^p < 0.01, ^***^p < 0.005, ^****^p < 0.001 *N* = 9–17 per strain per sex. Data represent mean ± SEM.

Lastly, CSS were examined individually using a between *Chrna5* genotype analysis for the effect of *Chrna5* genotype and sex on nicotine consumption. This analysis was to determine if the effect of *Chrna5* deletion on nicotine intake was eliminated relative to WT controls for each CSS. As shown in Figure 5 and Table 3, the effect of *Chrna5* deletion on nicotine intake was no longer significant in CSS1, CSS2, CSS5, CSS8, CSS9, CSS11, and CSS15. Three of these strains (CSS1, CSS5, and CSS11) also exhibited a main effect of sex. However, the lack of a significant genotype by sex interaction for any of these strains indicates that the effect of *Chrna5* genotype on nicotine consumption did not appreciably differ between female and male animals. For six strains (CSS3, CSS14, CSS16, CSS17, CSS19, and CSSX), there was a main effect for both genotype and sex suggesting the possibility that the effect of *Chrna5* genotype on nicotine intake might be sex dependent. Among these six strains, four (CSS14, CSS16, CSS19, and CSSX) exhibited modest sex differences in the effect of *Chrna5* genotype on intake. Nonetheless, the lack of a genotype by sex interaction suggests no meaningful difference of the effect of *Chrna5* genotype on nicotine consumption between female and male mice of these strains.

**Table 3:** Effect of *Chrna5* genotype and sex on B6 and individual CSS genetic backgrounds.

**CSS**	**Genotype**	**Sex**
B6	*F*_(1, 49)_ = 9.81, *P =* 0.003	*F*_(1, 49)_ = 0.792, *P =* 0.378
CSS1	*F*_(1, 44)_ = 2.95, *P =* 0.093	*F*_(1, 44)_ = 5.43, *P =* 0.024 [female: *F*_(1, 22)_ = 1.27, *P =* 0.272; male: *F*_(1, 22)_ = 2.59, *P =* 0.122]
CSS2	*F*_(1, 48)_ = 0.824, *P =* 0.369	*F*_(1, 48)_ = 1.39, *P =* 0.244
CSS3	*F*_(1, 44)_ = 26.51, *P =* 1 × 10^−6^	*F*_(1, 44)_ = 11.53, *P =* 0.001 [female: *F*_(1, 22)_ = 14.82, *P =* 8.7 × 10^−4^; male: *F*_(1, 22)_ = 11.71, *P =* 0.002]
CSS5	*F*_(1, 48)_ = 2.72, *P =* 0.106	*F*_(1, 48)_ = 6.24, *P =* 0.016 [female: *F*_(1, 23)_ = 0.738, *P =* 0.399; male: *F*_(1, 25)_ = 2.95, *P =* 0.099]
CSS6	*F*_(1, 46)_ = 14.66, *P =* 3.9 × 10^−4^	*F*_(1, 46)_ = 2.25, *P =* 0.140
CSS7	*F*_(1, 46)_ = 15.15, *P =* 3.2 × 10^−4^	*F*_(1, 46)_ = 0.532, *P =* 0.469
CSS8	*F*_(1, 46)_ = 1.125, *P =* 0.294	*F*_(1, 46)_ = 1.91, *P =* 0.173
CSS9	*F*_(1, 44)_ = 0.839, *P =* 0.365	*F*_(1, 44)_ = 1.75, *P =* 0.193
CSS10	*F*_(1, 43)_ = 4.09, *P =* 0.049	*F*_(1, 43)_ = 2.28, *P =* 0.139
CSS11	*F*_(1, 44)_ = 2.6, *P =* 0.148	*F*_(1, 44)_ = 1.10, *P =* 0.299
CSS12	*F*_(1, 44)_ = 17.64, *P =* 1.3 × 10^−4^	*F*_(1, 44)_ = 3.2, *P =* 0.081
CSS14	*F*_(1, 44)_ = 5.27 *P =* 0.027	*F*_(1, 44)_ = 9.34, *P =* 0.004 [female: *F*_(1, 22)_ = 1.7, *P =* 0.208; male: *F*_(1, 22)_ = 7.83, *P =* 0.01]
CSS15	*F*_(1, 58)_ = 1.35, *P =* 0.25	*F*_(1, 58)_ = 11.54, *P =* 0.001 [female: *F*_(1, 29)_ = 0.876, *P =* 0.357; male: *F*_(1, 29)_ = 0.556, *P =* 0.462]
CSS16	*F*_(1, 53)_ = 7.19, *P =* 0.010	*F*_(1, 53)_ = 5.9, *P =* 0.019 [female: *F*_(1, 24)_ = 1.84, *P =* 0.187; male: *F*_(1, 29)_ = 6.58, *P =* 0.016]
CSS17	*F*_(1, 48)_ = 34.14, *P =* 4.4 × 10^−7^	*F*_(1, 48)_ = 5.36, *P =* 0.025 [female: *F*_(1, 25)_ = 25.56, *P =* 3.2 × 10^−5^; male: *F*_(1, 23)_ = 10.41, *P =* 0.004]
CSS18	*F*_(1, 44)_ = 7.59, *P =* 0.008	*F*_(1, 44)_ = 0.02, *P =* 0.887
CSS19	*F*_(1, 44)_ = 7.25, *P =* 0.01	*F*_(1, 44)_ = 5.0, *P =* 0.03 [female: *F*_(1, 22)_ = 3.19, *P =* 0.088; male: *F*_(1, 22)_ = 4.59, *P =* 0.044]
CSSX	*F*_(1, 44)_ = 8.47, *P =* 0.006	*F*_(1, 44)_ = 4.95, *P =* 0.031 [female: *F*_(1, 22)_ = 4.57, *P =* 0.044; male: *F*_(1, 22)_ = 4.02, *P =* 0.058]

**Figure 6:**
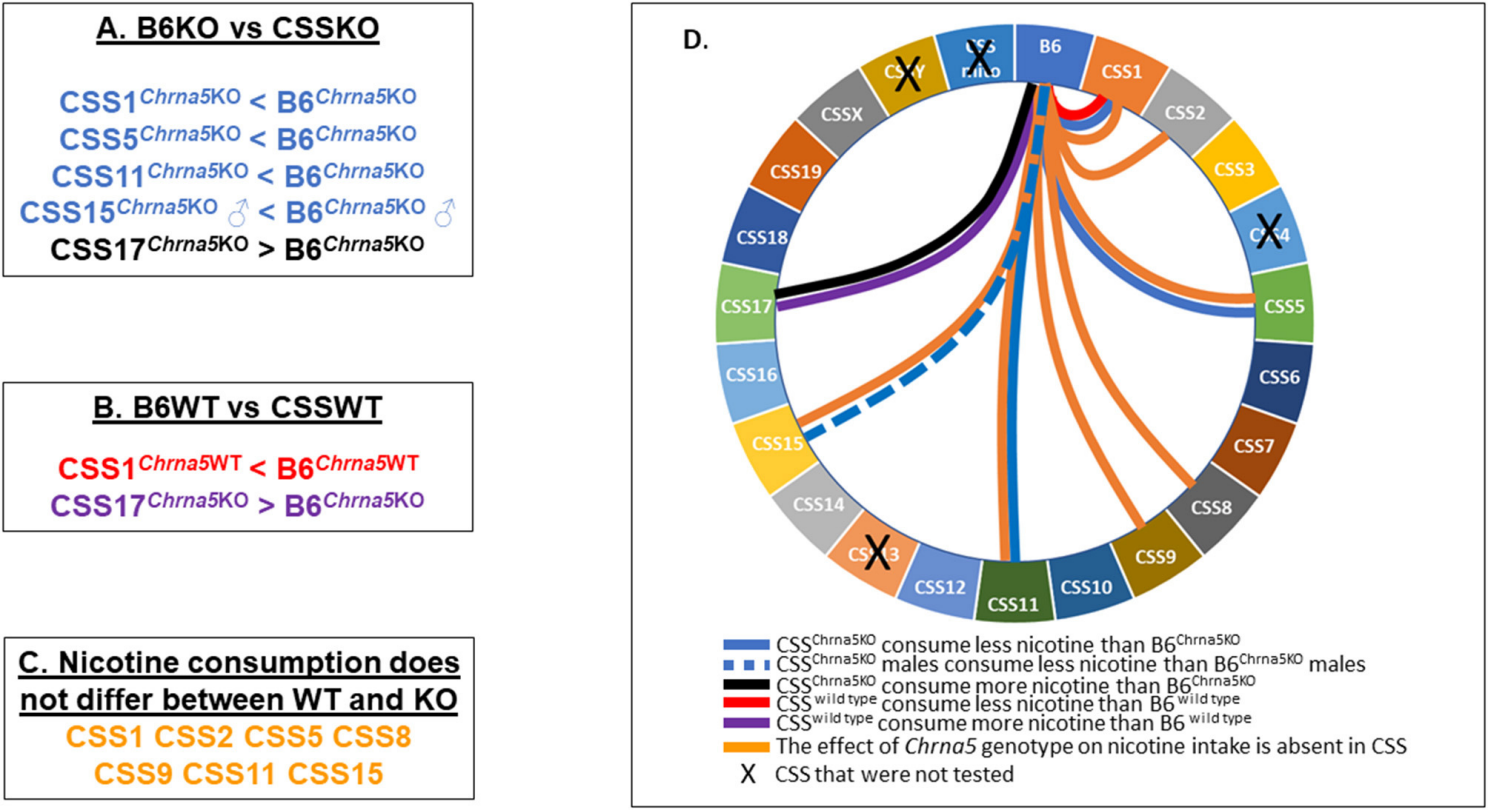
Summary of major findings. **(A)** Pairwise comparison between B6KO and CSSKO mice identified five CSSKO that differ in nicotine consumption from B6KO. **(B)** Pairwise comparison between B6WT and CSSWT identified two CSSWT that differ in nicotine consumption from B6WT. **(C)** Eight CSS were identified in which nicotine consumption did not differ between WT and KO mice. **(D)** Wheel diagram summarizing the overall results of the study.

In summary (Figure 6), amongst the eighteen CSS tested, six exhibited some effect on *Chrna5*-dependent nicotine consumption as indicated by either a within genotype (CSS1, CSS5, CSS11, CSS15) and/or between genotype comparison (CSS1, CSS2, CSS5, CSS8, CSS9, CSS11, CSS15), one strain (CSS17) impacted nicotine intake independent of *Chrna5* genotype and one strain (CSS1) altered nicotine intake in both a *Chrna5* genotype dependent and independent manner.

## Discussion

Previous studies have shown that deletion of *Chrna5*, the gene that encodes the nicotinic acetylcholine receptor subunit α5, significantly increases nicotine consumption (12, 13) in mice. The main goal of the current study was to utilize the C57BL/6J-Chr#^A^/J/NaJ (20, 23) chromosome substitution strains (CSS) to identify A/J chromosomes that possess potential genetic modifiers of the effect of *Chrna5* deletion on oral nicotine consumption. In principle, a genetic modifier is an allele of a gene that either diminishes, enhances or in some way alters the phenotypic effect of an allele of a different gene. Although not well-studied, genetic modifiers are common (18, 19), and when identified, can provide novel insight into molecular networks relevant to the mechanism through which a genetic perturbation impacts a phenotype as well as lead to the discovery of networks that can compensate for the genetic alteration of interest (19). Results of the current study indicate that A/J chromosomes 5, 11, and possibly 1 possess sex-independent modifiers of *Chrna5* null mutant-dependent nicotine intake and A/J chromosome 15 harbors a male-specific modifier. Evidence to support modifiers on CSS5, 11 and 15 (male only) includes the observation that nicotine intake in CSS5^*Chrna5*KO^, CSS11^*Chrna5*KO^, and male CSS15^*Chrna5*KO^ mice significantly differs from the reference B6^*Chrna5*KO^ strain. In all cases, the CSS^*Chrna5*KO^ animals consume less nicotine than B6*Chrna5*KO mice. In addition, mice that possess the WT allele of *Chrna5* in these three strains do not significantly differ from B6 mice harboring the WT allele indicating that the strain effect is specific to the *Chrna5* null allele. Further, the effect of *Chrna5* genotype on nicotine consumption in CSS5, CSS11, and male CSS15 mice is abolished (Table 3) indicating that the modifiers for each of these strains reduces nicotine intake in *Chrna5* null mutant mice to levels indistinguishable from WT mice. To the best of our knowledge, this is the first study to describe the mapping of genetic modifiers for any drug abuse-related trait.

Similar to CSS5^*Chrna5*KO^ and CSS11^*Chrna5*KO^ and male CSS15^*Chrna5*KO^, CSS1^*Chrna5*KO^ mice consume significantly less nicotine than do B6^*Chrna5*KO^ mice and the effect of *Chrna5* genotype on nicotine intake in CSS1 mice is abolished suggestive of a modifier on A/J chromosome 1. However, CSS1 WT mice also consume significantly less nicotine than do B6 WT mice. These results suggest the possibility that A/J chromosome 1 may possess a combination of alleles, one or more of which affects nicotine consumption in a genotype-independent manner while others act as a modifier in CSS1^*Chrna5*KO^ mice. Alternatively, the same allele or alleles may be responsible for the reduced nicotine intake in both the CSS1^*Chrna5*KO^ and CSS1 WT mice. In this case, the allele or alleles would have a greater effect on nicotine consumption in the CSS1^*Chrna5*KO^ mice relative to the CSS1 WT mice leading to the modifier-like effect. One other possibility is that there could be a floor effect on nicotine consumption in the CSS1 WT animals preventing a decrease in nicotine consumption similar to that seen in the CSS1^*Chrna5*KO^ mice. Future genetic dissection of chromosome 1 will be required to establish which of these possibilities is/are driving the effect of chromosome 1 on *Chrna5*-dependent and *Chrna5*-independent nicotine intake.

Three additional strains, CSS2, CSS8, and CSS9 show some evidence of possessing modifier alleles although they do not meet all criteria. The main factor that implicates these strains as potential modifier strains is the observation that nicotine consumption did not differ between WT and *Chrna5* null mutant mice in each of these three strains. However, neither the *Chrna5* null mutant or WT animals from these three strains differed significantly from the respective B6 reference control after controlling for an FDR of 0.05 or 0.1 making it somewhat difficult to interpret the modifier status of these three strains. Some support for a chromosome 2 modifier comes from a preliminary study that suggests that functional alleles of Chrna4, which differ between B6 and A/J mice (25, 28, 29) and are located on mouse chromosome 2, acts as a modifier of the *Chrna5* null mutation (12). Nonetheless, further studies will be required to determine whether CSS2 as well as CSS8 and CSS9 possess modifier alleles.

Although several A/J chromosomes were identified that possess genetic modifiers that eliminate the increase in nicotine consumption resulting from *Chrna5* deletion, it remains to be determined which gene or genes on each chromosome possess the nicotine consumption-modifying alleles. Once identified, these alleles should provide new insights into the molecular networks that can reverse the *Chrna5* deletion-dependent increase in nicotine consumption. Such knowledge may lead to a better understanding of the neurobiology of *Chrna5*-dependent nicotine consumption as well as suggest novel therapeutic strategies for treating nicotine dependence in humans.

One CSS that exhibited no evidence of a modifier but had a significant effect on nicotine consumption was CSS17. Both CSS17 WT and CSS17^*Chrna5*KO^ mice consumed significantly more nicotine than their respective B6 controls indicating that A/J chromosome 17 possess an allele or alleles that increases nicotine consumption independent of *Chrna5* genotype. In fact, CSS17 WT mice drank as much nicotine as did B6^*Chrna5*KO^ mice and CSS17^*Chrna5*KO^ mice consumed twice as much nicotine as did B6^*Chrna5*KO^ mice. Considering that B6 mice consume the highest level of nicotine among tested inbred strains and A/J mice are amongst the lowest nicotine consuming strains (25), it is somewhat surprising that A/J chromosome 17 harbors an allele or alleles of genes that nearly double the level of nicotine consumption in both the B6 WT and B6^*Chrna*5^ null mutant mice.

In the only other study that measured a nicotine response in the C57BL/6J-Chr#^A/J^/NaJ CSS panel, Boyle and Gill (30) assessed the effect of an acute injection of 1.5 mg/kg nicotine on locomotor activity. In this study, CSS17 mice, which showed an increase in locomotor activity following the administration of nicotine, significantly differed from B6 mice which displayed a decrease in activity to the same dose of nicotine. This finding suggests that A/J chromosome 17 either possesses separate alleles of genes that impact the acute effects of nicotine on locomotor activity and oral nicotine consumption independently or raises the possibility that there may be shared alleles on Chromosome 17 that affects both oral nicotine intake and the acute effects of nicotine on locomotion. The latter possibility suggests the potential for genetic overlap between these two nicotine measures. To assess a potential genetic overlap between the acute effects of nicotine on locomotion and oral nicotine intake, a correlational analysis between data extrapolated from Boyle and Gill (30) and data from CSS WT mice in the current study was performed. Comparing locomotor activity following an acute injection of 1.5 mg/kg nicotine to total μg/ml nicotine consumed across all common CSS tested between the two studies gave a non-significant but positive correlation of 0.328 (*P* = 0.183). Removing CSS9 from this analysis due to potential genetic differences from our CSS9 vs. the JAX CSS9 leads to a significant positive correlation (0.560, *P* = 0.019). Using dose (mg/kg/day) as an alternative measure of nicotine consumption in the CSS WT mice led to significant correlations with locomotor activity with (0.512, *P* = 0.03) or without (*r* = 0.666, *P* = 0.003) the inclusion of CSS9. In sum, these correlations are suggestive of a genetic overlap between oral nicotine intake and the acute effects of nicotine on locomotor activity with animals less sensitive to the locomotor depressant effects/more sensitive to the locomotor stimulating effects of nicotine consuming greater amounts of nicotine.

Genetic mapping of oral nicotine consumption in mice also has been reported in one previous study (31). In this study, which utilized an F2 intercross between B6 and C3H/HeJ mice, no quantitative trait loci (QTL) on chromosome 17 was detected for nicotine intake indicating that the association of chromosome 17 with nicotine consumption in the CSS panel is novel. Whether the association of chromosome 17 with nicotine intake in CSS17 mice and not the B6 and C3H/HeJ F2 intercross is the result of unique variants in A/J relative to C3H/HeJ, due to the distinct genetic structures of the two test populations or a consequence of the different designs of the nicotine consumption assays remains to be determined. Interestingly, a major QTL for nicotine consumption was detected on chromosome 1 in the previously published study. Whether the QTL identified on chromosome 1 in this study encompasses the same alleles that impact nicotine consumption independent of *Chrna5* genotype in CSS1 mice remains to be determined.

In summary, four CSS were identified that harbor apparent modifier alleles that eliminate the effect of *Chrna5* deletion on nicotine consumption and one CSS was identified that possesses an allele or alleles that substantially increases nicotine consumption independent of *Chrna5* genotype. Future studies with the CSS^*Chrna5*KO^ and CSS WT mice of interest can take advantage of the unique structure of the CSS (20, 23) to efficiently map the locations of the modifier loci and identify the specific genes/alleles on each of the A/J chromosomes that are responsible for the phenotypic effect on nicotine intake. These results should lead to a better understanding of the neurobiological mechanisms that drive nicotine consumption and potentially lead to the discovery of novel therapeutic targets for treating nicotine dependence.

## Limitations

At this time, we cannot entirely rule out the possibility that new, unidentified mutations in the B6 background of one or more of the CSS^*Chrna*5KO^ mice are responsible for the strain effect on the phenotype instead of the introgressed A/J chromosome. However, this possibility can be readily addressed in future studies by mapping the causal genomic region in a segregating population of CSS^*Chrna*5KO^ × B6 ^*Chrna*5KO^ mice.

## Data Availability Statement

The original contributions presented in the study are included in the article/Supplementary Material, further inquiries can be directed to the corresponding author/s.

## Ethics Statement

The animal study was reviewed and approved by University of Colorado Boulder Institutional Animal Care and Use Committee.

## Author Contributions

EM, ZW, and DW: animal husbandry, behavioral testing, genotyping, data entry, and database management. HM: data analysis and manuscript editing. JN and RR: experimental design, interpretation and manuscript, and figure editing. JS: experimental conception and design, data analysis, data interpretation, manuscript preparation and editing, and supervision of technical staff. All authors contributed to the article and approved the submitted version.

## Funding

This project was provided by NIH NIDA U01043802 (JS, RR, and JN).

## Conflict of Interest

The authors declare that the research was conducted in the absence of any commercial or financial relationships that could be construed as a potential conflict of interest.

## Publisher's Note

All claims expressed in this article are solely those of the authors and do not necessarily represent those of their affiliated organizations, or those of the publisher, the editors and the reviewers. Any product that may be evaluated in this article, or claim that may be made by its manufacturer, is not guaranteed or endorsed by the publisher.
